# Behavioral, physiological, and hormonal responses during pre-slaughter handling in goats: a comparison between trained and untrained handlers

**DOI:** 10.5713/ab.24.0050

**Published:** 2024-05-07

**Authors:** Pavan Kumar, Ahmed Abubakar Abubakar, Muideen Adewale Ahmed, Muhammad Nizam Hayat, Fakhrullah Abd Halim, Md. Moklesur Rahman, Mokrish Ajat, Ubedullah Kaka, Yong-Meng Goh, Awis Qurni Sazili

**Affiliations:** 1 Institute of Tropical Agriculture and Food Security, Universiti Putra Malaysia, 43400 UPM Serdang, Selangor, Malaysia; 2 Department of Livestock Products Technology, College of Veterinary Science, Guru Angad Dev Veterinary and Animal Sciences University, Ludhiana, Punjab 141004, India; 3 Department of Animal Science, Faculty of Agriculture, Universiti Putra Malaysia, 43400 UPM Serdang, Selangor, Malaysia; 4 Halal Products Research Institute, Universiti Putra Malaysia, Putra Infoport, 43400 UPM Serdang, Selangor, Malaysia; 5 Department of Veterinary Preclinical Studies, Faculty of Veterinary Medicine, Universiti Putra Malaysia, 43400 UPM Serdang, Selangor, Malaysia; 6 Department of Companion Animal Medicine and Surgery, Faculty of Veterinary Medicine, Universiti Putra Malaysia, 43400 UPM Serdang, Selangor, Malaysia

**Keywords:** Animal Handler, Physiological Responses, Preslaughter Handling, Training, Welfare

## Abstract

**Objective:**

The livestock handler attitude and their handling of animals is crucial for improving animal welfare standards, minimizing stress, improving productivity and meat quality. The present study was undertaken to assess the effect of training livestock handlers on behavioral, physiological, and hormonal responses during preslaughter handling in goats.

**Methods:**

A total of 6 handlers were divided into trained (trained in basic animal handling practices, animal behavior, and animal welfare), contact trained (not trained directly but interacted and saw the working of trained handlers), and untrained groups (no formal training). The handling experiment was conducted on 18 male goats by following a cross-over design. The goats were moved from lairage to slaughter point by trained, contact-trained, and untrained handlers. Various behavioral, physiological, and hormonal parameters were recorded at the lairage before handling and at the slaughter point after handling the goats.

**Results:**

The training of livestock handlers had a significant effect on behavioral, physiological, and hormonal responses in goats. The goats handled by untrained and contact-trained handlers were recorded with intense vocalization, significant (p<0.05) increase in heart rate and blood glucose, and catecholamines (adrenaline and nor-adrenaline), thereby indicating stress and poor animal welfare. The trained handlers were observed to use visual interactions (waving of hands or objects, blocking, hand raising, etc), and lower stress responses were recorded in the goats handled by this group.

**Conclusion:**

The present study highlights the importance of training to livestock handlers in improving animal welfare and minimizing stress in goats during pre-slaughter stress.

## INTRODUCTION

Various pre-slaughter handling practices and procedures of animals at the farm, during transport and marketing, and at the slaughterhouses may subject the animals to various stressors. These handling practices act as psychological or physical stressors and activate the sympathoadrenal (SPA) system as the first line of defense, resulting in the synthesis and release of catecholamines, consequently leading to ‘fight or flight behavior’ by these goats [1]. The hypothalamic-pituitary-adrenal axis is activated as a second line of defense if the stressor persists and affects various autonomous nervous systems, immune responses, and metabolism. All these changes resulted in behavioral, neural, hormonal, and musculoskeletal changes as a response to handling stress in animals [2], such as increasing excitability score, respiration rate, rectal temperature, and increased plasma cortisol and catecholamine concentrations [3,4], which in turn may negatively influence the carcass and meat quality attributes [5].

The attitude, behavior, and skills of livestock handlers are very important for minimizing preslaughter stress and improving meat quality in livestock during their handling [6]. Nielsen et al [7] noted that out of 40 identified hazards, nearly all (39 out of 40) originated from the handler’s mishandling of the animals. Proper training of livestock handlers is essential for good animal handling practices for inculcating positive attitudes and behavior toward animals [8]. There are several reports of inappropriate behavior and attitudes of livestock handlers resulting in the mishandling of animals during preslaughter handling [9].

The livestock handlers’ attitude and their handling of animals could be improved by proper and regular training on various aspects of proper animal handling and its role in animal welfare, improving meat quality, thereby countering negative narration of the meat industry. It would improve positive human-animal interactions by modifying behavior rather than skill transfer [10]. There are studies available on the role of behavioral/training interventions that resulted in altering the attitudes and behavior of workers [8,10].

There is a lack of studies on the effect of training livestock handlers on behavioral, physiological, and hormonal responses during the crucial stage of preslaughter handling in goats i.e., from lairage to slaughter point. Thus, the present study was designed to assess the effects of training livestock handlers on behavioral, physiological, and hormonal responses during preslaughter handling in goats. The outcome of the present study would be helpful in convincing the people involved in the meat industry to follow proper animal handling principles.

## MATERIALS AND METHODS

### Ethical approval

The present study was conducted following the animal ethics guidelines of the Research Policy of Universiti Putra Malaysia as per Institutional Animals Care and Use Committee approval No.: UPM/IACUC/AUP-R003/2022, dated 27 May 2022.

### Animals

A total of 18 goats (male, Boer cross, 8 to 12 months of age, 24 to 28 kg live weight) were used in the present study. The animals were housed and acclimatized at the Small Ruminant Housing Facility at the Institute of Tropical Agriculture and Food Security in Universiti Putra Malaysia (latitude 2°59′06.5″N and longitude 101°43′40.7″E) for 14 days. Animals were fed twice daily and had *ad libitum* water. Throughout their stay, physiological parameters (heart rate, rectal temperature, breathing, feeding, and activity) were recorded daily on the animal monitoring sheet. Animals had proper access to veterinary services. Prior to the start of the experiment, the animals were transported (2.0 km; 30 to 40 min in loading, transit, and unloading) to the research slaughterhouse of the Department of Animal Science, Faculty of Agriculture, Universiti Putra Malaysia (258059.000″ N; 10144006.400″ E). The animals rested overnight in the lairage with *ad libitum* water.

### Experimental design

This study was conducted in July–August 2022. Six livestock handlers involved in the routine operation at the farm (age of 25 to 28 years old; education level, matriculation to graduate) were selected for the study, out of which, 2 handlers were trained in basic behavioral principles of handling goats such as gentle handling, animal flight zone, point of balance, normal movement, stress responses, and their consequences (T group), and 2 handlers have not undergone training for these principles (UT, untrained). The remaining 2 handlers were not directly trained but interacted with trained handlers and observed the working of trained handlers (CT, contact trained staff) for a period of 1 week. After the completion of the training session, the attitude and skill of trainees were evaluated through oral viva and practical examination. The trainees graded good - excellent were included for animal handling experiments as trained handlers. Before the beginning of the experiment, the trained handlers were again briefed about handling practices and animal behavior to refresh their knowledge. The remaining 2 handlers were not directly trained but rather, interacted with the trained handlers and observed the working of trained handlers (CT, contact trained staff). The interaction of handlers and goats was assessed during preslaughter handling in terms of measuring behavioral responses, physiological responses, stress hormones, and blood parameters.

A total of 18 goats (similar age group, breed, and live weight) were used in the study by following a crossover design. Animals were allotted to three groups, with each group having 6 animals and crossed over three times (n = 18) during the study as per Table 1. A one-week interval was maintained in between trials to neutralize the treatment effect in goats.

Goats were moved from the lairage to the slaughter point at the same distance and floor conditions by the animal handlers. The welfare status of goats to handling by trained staff (T), contact trained (CT), and untrained staff (UT) were assessed by recording behavioral parameters (vocalization, escape behavior, turning backward, and involuntary urination), physiological parameters (heart rate, rectal temperature, and blood glucose), and hormonal responses (catecholamines and β-endorphin), before handling (in lairage) and after handling (at slaughter point). The preslaughter handling of goats was recorded by noting the human-animal interactions by tactile (handling of goats by using ear, legs, tail, hitting, prodding, wooden sticks, etc), auditory (whistling, shouting, banging of pen fittings, etc) and visuals means (waving of hands or objects in front of goats, blocking, hand raising, etc).

### Behavioral responses

The behavior of the goats was recorded manually through video recording (close-circuit television cameras; Reolink Ltd, Wan Chai, Hong Kong Island) of the movement of the goats during the experiment. These parameters were recorded by two technical staff members with expertise in goat handling and animal welfare. Vocalization was recorded by measuring the number of bleats and their intensity/pitch. The behavioral observation included escape behavior (avoiding stressors), frequency and intensity of vocalization, and involuntary urination. Urination was recorded by counting the number of times the goat urinated during the experiment.

The behavioral scores of goats for vocalization, escape be havior, and turning backward during handling were scored on a 0 to 3 point scale, where a score of 0 was awarded when the goat remained calm, and the particular behavior response was absent, a score of 1 when the response of the goat was mild, a score of 2 when the response of the goat was medium and a score of 3 when the response of the goat was intense. For involuntary urination, a scale of absent (−) and present (+) was used. The total percentages of goats showing particular behavioral responses were recorded as frequency (Table 2).

### Physiological responses

The physiological responses of animal handlers in goats were measured by recording heart rate (by stethoscope), rectal temperature (by thermometer), and blood glucose (by portable blood glucometer by putting a drop of blood on a test strip onto the device) at the lairage before handling and at the slaughter point after handling. During the process of data recording and blood collection, goats were restrained minimally, and heart rate measurement and blood collection were performed by experienced technical staff.

### Blood collection and hormonal analysis

Blood collection was performed by the trained technical staff from the external jugular vein. Blood samples were collected at two points, viz. before handling in lairage (BH) and at slaughter point after handling (AH). Blood samples were collected by using 21-gauge needles connected to a 10 mL clot activator (BD Vacutainer, Plymouth, UK) ethylenediaminetetraacetic acid (EDTA) tubes. The tubes containing collected blood samples were kept slat in a box containing crushed ice for 1 h, followed by refrigerated centrifugation (Eppendorf Centrifuge 5810) at 3,500 g for 15 min at 4°C. The plasma obtained was stored in a deep freezer (Sanyo Electric Co Ltd, Osaka, Japan) at −80°C, until subsequent hormonal analysis.

Plasma concentrations of catecholamines and β-endorphin were determined using the highly sensitive adrenaline (BA E-4100), noradrenaline (BA E-4200), and β-endorphin (QY-E140008) enzyme-linked immunoassay kits (ELISA; QAYEE-BIO-Technology Co. Ltd, Shanghai, China) following the manufacturer’s instructions. The competitive ELISA kits used the microtiter plate format, where the hormones were extracted from a plasma sample using a cis-diol-specific affinity gel, acylated, and then modified enzymatically. The antigen was bound to the solid phase of the microtiter plate and the derivatized standards, controls, samples as well as the solid phase bound analytes compete for a fixed number of anti-serum binding sites.

### Statistical analysis

The data were tested for normal distribution using a Shapiro–Wilk test using SPSS Statistics Version 20 software (IBM Corporation, New York, USA). The data were presented as mean along with standard error. The present study used a cross-over design by dividing 18 goats into 3 groups (trained, contact trained, and untrained groups with 6 goats per group) and crossing over three times (n = 18 as 6 goats in a group ×3 times handling experiment). A paired t-test was used to analyze the differences between physiological and hormonal responses for before-handling values (measured at lairage before the start of the handling experiment) and after-handling values (measured after handling at the slaughter point) of each group handled by trained, contact trained, and untrained handlers.

Further, differences among groups after handling at slaugh ter point (viz., trained group, goats handled by trained handler; contact trained group, goats handled by contact trained handlers; and untrained group, goats handled by untrained handlers) and pooled control (goats prior to handling in lairage) values in lairage were compared with Duncan’s multiple-range test by using a one-way analysis of variance (ANOVA, n = 18). Responses between groups and handling intensities were compared with Duncan’s multiple-range test by using ANOVA. A level of significance (p-value) of less than 0.05 was considered statistically significant.

## RESULTS AND DISCUSSION

The animal handlers’ attitudes and empathy have a major impact on animal stress and welfare. This attitude and empathy of animal handlers are largely affected by the behavior and training of the animal handlers [11]. Thus, assessing the animal response to handling (human-animal interactions) during preslaughter handling indicates the attitude and empathy of animal handlers.

### Behavioral responses

The pre-slaughter handling had a significant effect on the behavioral response of the goats. The training of livestock handlers had a significant (p<0.05) effect on behavioral response in goats (Table 2). The goats exhibited intense vocalization and escape behavior upon handling (mobilized) by the untrained handler. Involuntary urination was also recorded in goats handled by untrained handlers. While moving from the lairage to the slaughter point, goats spent a significant amount of time fleeing, vocalizing, trying to escape, or turning around. This behavior was positively correlated with heart rate, blood glucose level, and temperature, suggesting that goats that consistently exhibited these ostensibly avoidance or fear behaviors were at risk of causing injuries, poor carcass, and meat quality [12].

Interaction with humans has a significant impact on the behavior and physiology of domestic animals such as goats [13]. Rough handling can negatively affect animal welfare and increase fear in animals towards humans. Good practice of preslaughter handling of beef cattle was recorded to have a positive impact on animal behavior and production [8]. Vocalization during preslaughter handling is related to the physiological measures of stress or painful process. Vocalization is considered an indicator of the welfare of goats, where a higher intensity of vocalization usually indicates a sigh of distress and fear [14]. Grandin and Vogel [15] observed higher vocalization scores in slaughterhouses linked with poor animal handling, restraints, and stunning practices; thereby using vocalization scores as an animal welfare indicator in cattle and pig slaughterhouses.

Exposure to stress has been documented to increase the micturition frequency and overactive bladder conditions due to the activation of peripheral and central nervous systems. The release of pro-micturition molecules (such as BB-like peptides, AngII, and nitric oxide) in the brain under exposure to stress could also increase the urination frequencies [16].

### Physiological responses

The livestock handlers’ attitude significantly affected the physiological responses in goats during preslaughter handling (Table 3). While analyzing various physiological responses within the groups, the BH value of heart rate was recorded significantly (p<0.05) lower than the AH value in goats handled by untrained handlers and contact-trained handlers. The before and after handling values of the heart rate of goats handled by trained handlers were recorded as comparable (p>0.05). The before and after handling values of rectal temperature were comparable in goats handled by trained and contact-trained handlers. The after-handling values of rectal temperature were recorded significantly (p = 0.007) higher as compared to their respective after-handling values in goats handled by untrained trainers. The after-handling blood glucose levels recorded a marginal increase (p>0.05) as compared to their respective before-handling values among all three groups of goats handled by trained, contact-trained, and untrained handlers.

Further, while analyzing physiological responses within groups after handling with control values in lairage before handling, the heart rate was recorded as highest and significantly (p<0.05) higher in goats handled by CT, whereas the mean heart rate was recorded comparable for the control and goats handled by trained group (Table 4). Rectal temperature was recorded as significantly higher in goats handled by UT as compared to control, whereas the rectal temperature for goats handled C, T, and CT was recorded with a non-significant difference (p>0.05). Blood glucose values for goats handled by CT and UT, and C and T were recorded as non-significant (p>0.05); however, goats handled by CT and UT groups had significant (p<0.05) higher values as compared to goats handled by C and T.

An increase in catecholamines and glucocorticoids under stress in goats results in higher heart rate and blood glucose levels by increasing glucose production from glycogenolysis and gluconeogenesis required for preparing animals for the response to a stressor (fight or flight response) [5]. Kumar et al [17] also reported an increase in the blood glucose and heart rate in goats under psychological stress during the slaughtering of goats. An increase in the blood glucose value in goats is an indicator of stress in goats [18].

The nature of stress also affects the physiological respons es in animals, with the untrained group having aversive human-animal interactions, leading to more pronounced physiological responses. These untrained handlers handled goats by using unacceptable methods not permitted under various animal welfare principles and laws. The trained group handled animals minimally and gently, hence pronouncing lower stress response and better compliance with animal welfare. Overall stress response by an animal is multivariant and governed by various factors and their interactions such as types, duration, intensity, previous exposure, temperament, etc. Handling of animals by different methods initiates different stress responses in animals, such as pigs handled by electric goads and by nose snare for 5 minutes resulting in higher heart rates in pigs moved by electrical goads [19].

### Hormonal responses

**Figure 1:**
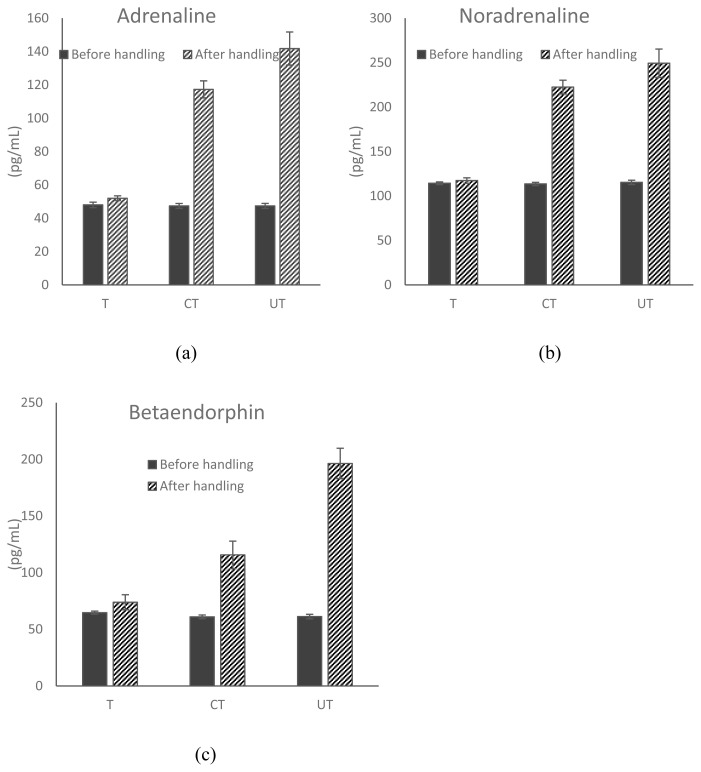
The before-handling and after-handling values of (a) adrenaline, (b) noradrenaline, and (c) beta-endorphin, hormones in goats affected by trained, contact-trained, and untrained handlers, respectively. Values are mean±standard error. T CT and UT represent the respective hormonal value in goats handled by trained, contact trained, and untrained handlers, n = 18.

Livestock handler training had a significant impact on the hormonal responses in goats (Figure 1a, 1b, 1c). While analyzing various hormonal responses within the groups, the mean values for blood catecholamines (adrenaline and nor-adrenaline) and β-endorphin before and after handling were recorded as higher compared to those in goats handled by trained handlers. For contact-trained and untrained groups, adrenaline values after handling were 2.48 and 3.0-fold higher, respectively, than their corresponding values before handling. Similarly, the values for nor-adrenaline were 1.96, and 2.16-fold higher after handling for contact-trained and untrained groups than before-handling values. For β-endorphin, these values were 1.90 and 3.20-fold higher for contact-trained and untrained groups after handling compared to before-handling values.

Further, while analyzing hormonal responses within groups after handling with control values in lairage before handling, the mean values of adrenaline and β-endorphin for C and T goats were recorded as comparable (p>0.05), but significantly (p<0.05) lower than their respective CT values (Table 5). Further, adrenaline and β-endorphin values were reported significantly (p<0.05) higher in UT as compared to their CT values. The nor-adrenaline value was recorded as comparable in CT and U goats.

In the present study, handling stress due to a potentially aversive attitude leading to negative human-animal interactions initiated short-term stress responses in goats, thereby activating the SPA axis. This SPA activation caused the release of neurotransmitter catecholamines into the circulatory system to adjust homeostasis and organize the body to situations warranting high energy production. Further, a majority of the noradrenaline present in the blood is due to diffusion from the noradrenergic nerve endings of SPA into the blood [20]. An increase in adrenaline concentration after handling in CT and UT goats could be due to demanding situations of higher energy expenditure and preparedness for a fight or flight response of the autonomous nervous system. Similarly, an increase in catecholamines (adrenaline and nor-adrenaline) concentration under preslaughter stress (transportation) was reported in goats [21] in lamb [22]. A significant increase in the plasma catecholamines (adrenaline and nor-adrenaline) concentrations in goats upon slaughter has been reported by Sabow et al [23].

β-Endorphins are peptide hormones released by the hy pothalamus and pituitary glands in response to stress or pain to relieve pain and create a general feeling of well-being. However, an increase in plasma β-endorphin concentration indicates more about stressful conditions rather than pain [24]. A rapid increase in β-endorphin value was also reported in ewes and rams under restrain stress, tail docking, and castration by Mears and Brown [25] and recommended assessment of β-endorphin as an important stress indicator in lamb. A rapid influx of β-endorphin (5 to 10 times) in male rats upon psychological stress of (40 to 60 dB sound) [26] and immobilization [27]. However, as the blood circulatory β-endorphin has not seemed to reach the central nervous system, thus plasm β-endorphin is widely correlated with stress rather than pain perception [28].

## CONCLUSION

The present study highlighted the importance of proper training for animal handlers in animal handling and welfare principles. The training of livestock handlers was observed to improve their attitude and behavior, thereby avoiding negative human-animal interactions as reflected by the behavioral responses, physiological responses, catecholamines, and β-endorphins. However, in the present study, animal handling has marginal improvement by contact-trained handlers as compared to trained handlers, but it was not significant. Further, the handling of goats by the untrained and contact-trained group was done mostly by tactile interaction by leg, horn, and ear; which are not permitted under animal welfare regulations. Thus, proper training is recommended for livestock handlers during the preslaughter handling of animals in slaughterhouses.

## LIMITATIONS AND PROSPECTS

The behavior of animal handlers and their interaction with animals leading to forceful handling of animals are affected by various factors such as lack of control of their actions, time constraints, workload, poor infrastructure, and inappropriate/lack of knowledge about animal handling and welfare. One welfare concept is the interrelation between animal welfare to human well-being, the environment, and biodiversity. The personality of stockpersons, such as the tough-mindedness and cognitive status of the stockpersons, should also be taken into consideration as it has a direct effect on the outcome of training. The overall stress response in animals is complex and multivariant depending upon the stress types, duration, and intensity; animal breed, age, temperament, microclimate, and prior exposure to stressor. This results in variable outcomes of stress responses in animals. Various factors, such as the novelty of the environment and separation from the social group, also cause stress and fear in goats, thereby affecting the stress responses. Further prolonged acute exposure also leads to stress adaptation in animals, with animals showing chronic stress responses.

There should be proper mechanisms to regularly evaluate the impact of training sessions. The training outcome or the impact of training on livestock handlers’ attitudes and behavior depends upon the cognitive status, temperament, and empathy of the trainees. Further, the jobs in the livestock sectors, especially during the slaughtering process are very tedious and stressful where the personnel are working at high speed to match the slaughter rate, monotonous, and under low temperatures. Thus, to ensure proper human and animal welfare by adhering to ‘One Welfare’ concept, there should be the provision of continuous monitoring, motivating the animal handlers to follow proper animal handling principles, with regular financial and non-financial incentives/rewards given for them for following and adhering to these principles of animal handling.

## Figures and Tables

**Table 1:** Cross-over design used for the study

Experiment	Animals		

	T	CT	UT
First trial	A1–A6	A7–A12	A13–A18
Second trial	A13–A18	A1–A6	A7–A12
Third trial	A7–A12	A13–A18	A1–A6

T, goats handled by trained handlers; CT, goat handled by handler not trained but interacted and saw the handling of trained handlers; UT, goats handled by the untrained handler, A-goat.

**Table 2:** Effect of training of livestock handlers on various behavioral parameters during preslaughter handling in goats

Parameters	Trained		Contact training		Untrained	
		
	Frequency (%)^1)^	Scale^2)^	Frequency (%)^1)^	Scale^2)^	Frequency (%)^1)^	Scale^2)^
Vocalization	0	0	75	3	100	3
Turning backward	0	0	0	0	100	1
Escape behavior	25	1	75	2	100	3
Urination	0	-	0	-	50	+

1) Frequency-number of animals showed the behavioral responses under handling stressors.

2) Scale for vocalization, turning backward, and escape behaviour: 0, absence of response; 1, mild response; 2, medium response; 3, intense response.

Scale for urination: −, as absent; +, as present.

**Table 3:** Physiological responses in goats before and after handling by trained, contact-trained, and untrained handlers

Group	Heart rate (beats/min)			Temp (Rectal, °C)			Blood glucose (mMol/L)		
		
	BH	AH	p-value	BH	AH	p-value	BH	AH	p-value
Trained (T)	71.70±1.22	73.25±1.38	0.062	37.36±0.18	37.64±0.13	0.142	3.92±0.08	3.94±0.10	0.921
Contact trained (CT)	72.25±0.99	109.40±3.24	0.003	37.23±0.11	37.87±0.18	0.117	3.94±0.08	4.51±0.10	0.657
Untrained (UT)	71.90±0.97	120.35±5.52	0.016	37.23±0.11	38.08±0.21	0.007	3.90±0.06	4.58±0.11	0.192

Values are mean±standard error, n = 18.

BH, value before handling in lairage; AH, value after handling at slaughter point.

**Table 4:** Physiological responses in goats affected by handling by trained, contact trained and untrained handlers

Parameter	Control	Trained	Contact trained	Untrained
Heart rate (beats/min)	71.70±1.22^a^	73.25±1.38^a^	109.40±3.24^b^	120.35±5.52^c^
Temp (Rectal, °C)	37.36±0.18^a^	37.64±0.13^ab^	37.87±0.18^ab^	38.08±0.21^b^
Blood glucose (mMol/L)	3.89±0.08^a^	3.92±0.09^a^	4.52±0.09^b^	4.58±0.10^b^

Values are mean±standard error with different superscripts within a row-wise differ significantly (p<0.05); control-values recorded at lairage before handling, n = 18.

a–c Means in a row with common letters differ (p<0.05).

**Table 5:** Hormonal responses in goats handled by trained, contact-trained, and untrained handlers

Hormone	Control	Trained	Contact trained	Untrained
Adrenaline (pg/mL)	48.01±1.65^a^	51.96±1.46^a^	117.28±5.12^b^	141.73±9.95^c^
Nor-adrenaline (pg/mL)	89.71±7.83^a^	117.39±3.03^b^	222.49±7.74^c^	249.29±15.89^c^
β-endorphin (pg/mL)	64.66±1.37^a^	73.94±6.61^a^	115.69±12.14^b^	196.37±13.36^c^

Values are mean±standard error with different superscripts within a row-wise differ significantly (p<0.05); control-values recorded at lairage before handling, n = 18.

a–c Means in a row with common letters differ (p<0.05).
